# Metabolomic profiling in kidney cells treated with a sodium glucose-cotransporter 2 inhibitor

**DOI:** 10.1038/s41598-023-28850-3

**Published:** 2023-02-04

**Authors:** Hyung Ah Jo, Jong-Hyun Seo, Sunhwa Lee, Mi-yeon Yu, Eunjin Bae, Dong Ki Kim, Yon Su Kim, Da Jung Kim, Seung Hee Yang

**Affiliations:** 1 Department of Internal Medicine, Inje University Ilsan Paik Hospital, Goyang, Korea; 2 Metabolomics Core Facility, Department of Transdisciplinary Research and Collaboration, Biomedical Research Institute, Seoul National University Hospital, 103 Daehak-ro, Jongno-gu, Seoul, Korea; 3 Department of Clinical Medical Science, Seoul National University, Seoul, Korea; 4 Department of Internal Medicine, Kangwon National University Hospital, Chuncheon, Gangwon-Do Korea; 5 Department of Internal Medicine, Hanyang University Guri Hospital, Guri, Korea; 6 Department of Internal Medicine, Institute of Health Science, College of Medicine, Gyeongsang National University, Gyeongsang National University Changwon Hospital, Jinju, Korea; 7 Department of Internal Medicine, Seoul National University Hospital, Seoul, Korea; 8 Department of Internal Medicine, Seoul National University College of Medicine, Seoul, Korea; 9 Biomedical Research Institute, Seoul National University Hospital, 103 Daehak-ro, Jongno-gu, Seoul, Korea; 10 Kidney Research Institute, Seoul National University, 103 Daehak-ro, Jongno-gu, Seoul, Korea

**Keywords:** Cell biology, Nephrology

## Abstract

We aimed to determine the metabolomic profile of kidney cells under high glucose conditions and following sodium-glucose cotransporter 2 (SGLT2) inhibitor treatment. Targeted metabolomics using the Absolute IDQ-p180 kit was applied to quantify metabolites in kidney cells stimulated with high glucose (25 and 50 mM) and treated with SGLT2 inhibitor, dapagliflozin (2 µM). Primary cultured human tubular epithelial cells and podocytes were used to identify the metabolomic profile in high glucose conditions following dapagliflozin treatment. The levels of asparagine, PC ae C34:1, and PC ae C36:2 were elevated in tubular epithelial cells stimulated with 50 mM glucose and were significantly decreased after 2 µM dapagliflozin treatment. The level of PC aa C32:0 was significantly decreased after 50 mM glucose treatment compared with the control, and its level was significantly increased after dapagliflozin treatment in podocytes. The metabolism of glutathione, asparagine and proline was significantly changed in tubular epithelial cells under high-glucose stimulation. And the pathway analysis showed that aminoacyl-tRNA biosynthesis, arginine and proline metabolism, glutathione metabolism, valine, leucine and isoleucine biosynthesis, phenylalanine, tyrosine, and tryptophan biosynthesis, beta-alanine metabolism, phenylalanine metabolism, arginine biosynthesis, alanine, aspartate and glutamate metabolism, glycine, serine and threonine metabolism were altered in tubular epithelial cells after dapagliflozin treatment following 50 mM glucose compared to those treated with 50 mM glucose.

## Introduction

Sodium-glucose cotransporter-2 (SGLT2) inhibitors block the sodium-glucose transport protein 2 from acting in the early proximal tubule, which is responsible for the majority of glucose reabsorption in the kidney, resulting in a glucose-lowering effect via glycosuria^1^. SGLT2 inhibitors have been extensively studied in a number of clinical trials aimed at improving cardiovascular outcomes and providing renal protection in individuals with type 2 diabetes mellitus^2–6^. SGLT2 inhibitors not only have a glycosuric impact but also restore tubuloglomerular feedback by decreasing the delivery of sodium and glucose to the distal tubule, thereby reversing afferent vasodilatation. These processes restore the increased glomerular pressure that plays a detrimental role in the progression of diabetic nephropathy^7^. This pleiotropic action of SGLT2 inhibitors beyond their glucose-lowering effect can influence a number of kidney cells as well as tubular cells on which SGLT2 inhibitors primarily act^8^. Diabetic nephropathy is caused by complicated cellular signaling and the paracrine effect of kidney cells including podocytes, tubular epithelial cells, and glomerular endothelial cells^9^.

A comprehensive metabolomics study at the cellular level can shed light on the distinct biological responses of kidney cells to the diabetic milieu^10^. Although the metabolomic study used urine samples in patients with diabetes mellitus who had taken SGLT2 inhibitors^11^, the metabolomics of kidney cells under high glucose conditions and with SGLT2 inhibitors have not been investigated. In the present study, we aimed to determine the metabolomic profile of kidney cells under high glucose conditions and following treatment with an SGLT2 inhibitor based on several clinical studies that have revealed promising results regarding the renoprotective effect of SGLT2 inhibitors^5,6^. Targeted metabolomics using the Absolute IDQ-p180 kit was applied to quantify 188 metabolites in kidney cells stimulated with high glucose (25 and 50 mM) and treated with the SGLT2 inhibitor, dapagliflozin.

## Materials and methods

### Ethics statements and patient tissue collection

This study using cultured primary human kidney cells was conducted and approved by the institutional review board of Seoul National University Hospital, Seoul, Korea (H-2112-042-1280). Kidney sections were obtained from the distal part of nephrectomy specimens from patients with renal cell carcinoma. Informed consent was obtained from all subjects and their legal guardians. All methods were carried out in accordance with relevant guidelines and regulations. The culture methods for each kidney cell line are as follows. Cells (2 × 10^5^/well) were then placed in 6-well chamber slides with serum-free medium for 24 h.

### Primary cultured human tubular epithelial cells

The kidney cortex was dissected and the minced specimens were digested with Hank’s balanced salt solution with 3 mg/mL collagenase (Sigma‒Aldrich, St. Louis, MO) and were incubated at 37 °C for 1 h. The cells were washed with phosphate buffered saline (PBS) through a serial sieving, and centrifuged at 500 × *g* for 5 min. The cells were incubated in DMEM/F12 (Lonza, Walkersville, MD) for 4–6 h and floating tubular cells in the media were collected and cultured on collagen-coated dishes (BD Biosciences, San Jose, CA) until epithelial cell colonies were established. To identify tubular epithelial cells, a fluorescence-activated Cell Sorting Calibur instrument (BD Biosciences) with FITC-labeled anti-AQP1 staining (Abcam, Cambridge, MA) was used at 4 °C for 30 min^12,13^.

### Primary cultured human podocytes

The dissected kidney cortex was used, and isolated glomeruli were cultured for 8 days. The outgrowing cells were trypsinized and passed through serial sieves. The cells were washed with PBS and incubated with Fc receptor blocking reagent (1 μg/mL, BD Bioscience). To identify podocytes, the PE-conjugated rabbit anti-human-purified anti-podocalyxin (R&D Systems, Minneapolis, MN) was used. The media consisted of DMEM/F12 (Lonza) supplemented with 15% fetal bovine serum (FBS, Gibco, Thermo Fisher Scientific, Waltham, MA), 1 × insulin-transferrin-selenium (Gibco), HEPES buffer (10 mmol, Sigma‒Aldrich), L-glutamine (200 μmol, Gibco), hydrocortisone (50 nmol, Sigma‒Aldrich), penicillin (100 U/mL, Gibco) and streptomycin (100 pg/mL, Gibco) and was changed every 3 days. Plating was performed on plastic dishes coated with fibronectin (10 μg/mL, Sigma‒Aldrich)^14,15^.

### In vitro experiments

Primary cultured human kidney cells were stimulated with 25 mM or 50 mM glucose and the cells were harvested at 24 h to further metabolomic analysis. Cells were treated with 50 mM glucose followed by 2 μM of dapagliflozin, and cells were harvested 24 h later to investigate the metabolomic profile change following dapagliflozin treatment.

### Metabolomic analysis

The AbsoluteIDQ p180 kit with an LC‒MS/MS system (Sciex 6500+ QTRAP) was used to quantify 188 metabolites, allowing the concurrent high-throughput detection and quantification of metabolites in primary cultured human kidney cells. The primary cultured cells were lysed by freezing and thawing on ice and were then resuspended in ice-cold absolute ethanol/0.01 M phosphate buffer. The samples were sonicated in an ice-cold water bath for 3 min and the cell lysates resuspended in the buffer were centrifuged at 2 °C and at 31,500 × *g* for 5 min. The supernatants were harvested and used for the p180 kit. The sample preparation and LC‒MS/MS analytical procedure are described in detail in previous reports^16,17^.

### Statistical analysis

Metaboanalyst Version 5.0 was utilized for conducting sparse partial-least-squares discriminant analysis (sPLS-DA), pathway analysis, and visualizing heatmap. Data were compared using Graphpad Prism 9.0 (GraphPad Software Inc., San Diego, CA, USA) between control, 50 mM glucose treated, and 50 mM glucose treatment followed by 2 μM dapagliflozin-treated samples of primary cultured human tubular epithelial cells, and podocytes. Logarithmic transformation (base 2) was adopted to assess the magnitude of fold difference observed in metabolites concentration under high glucose condition (50 mM of glucose) compared with control or treatment with 2 μM of dapagliflozin following high glucose condition (50 mM of glucose). Statistical significance was determined as *p* < 0.05 using the unpaired nonparametric Mann–Whitney test. MetaboAnalyst ver 5.0 (https://www.metaboanalyst.ca) was utilized to visualize heatmaps. Heatmaps with group average of significant metabolites in kidney cells were generated using autoscale feature standardization, Elucidean distance measure, and Ward’s clustering algorithm.

## Results

### The metabolomic pattern of kidney cells treated with an SGLT2 inhibitor following high glucose stimulus

**Figure 1:**
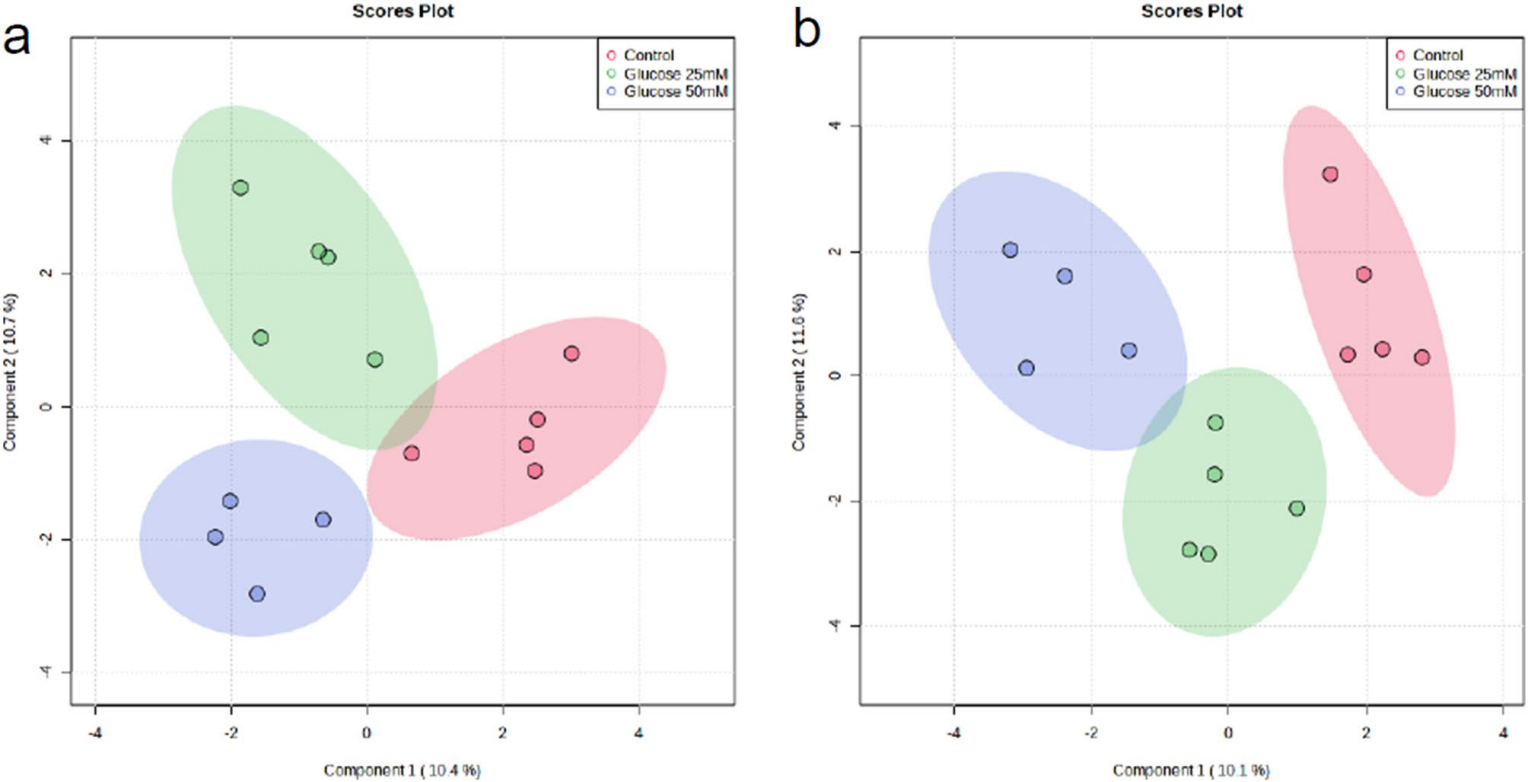
Sparse partial-least-squares discriminant analysis (sPLS-DA) of metabolites detected in tubular epithelial cells (**a**), podocytes (**b**), respectively, treated with glucose (25, 50 mM) compared to those without treatment (control). sPLS-DA in 2D scoring plot with 95% confidence interval.

**Figure 2:**
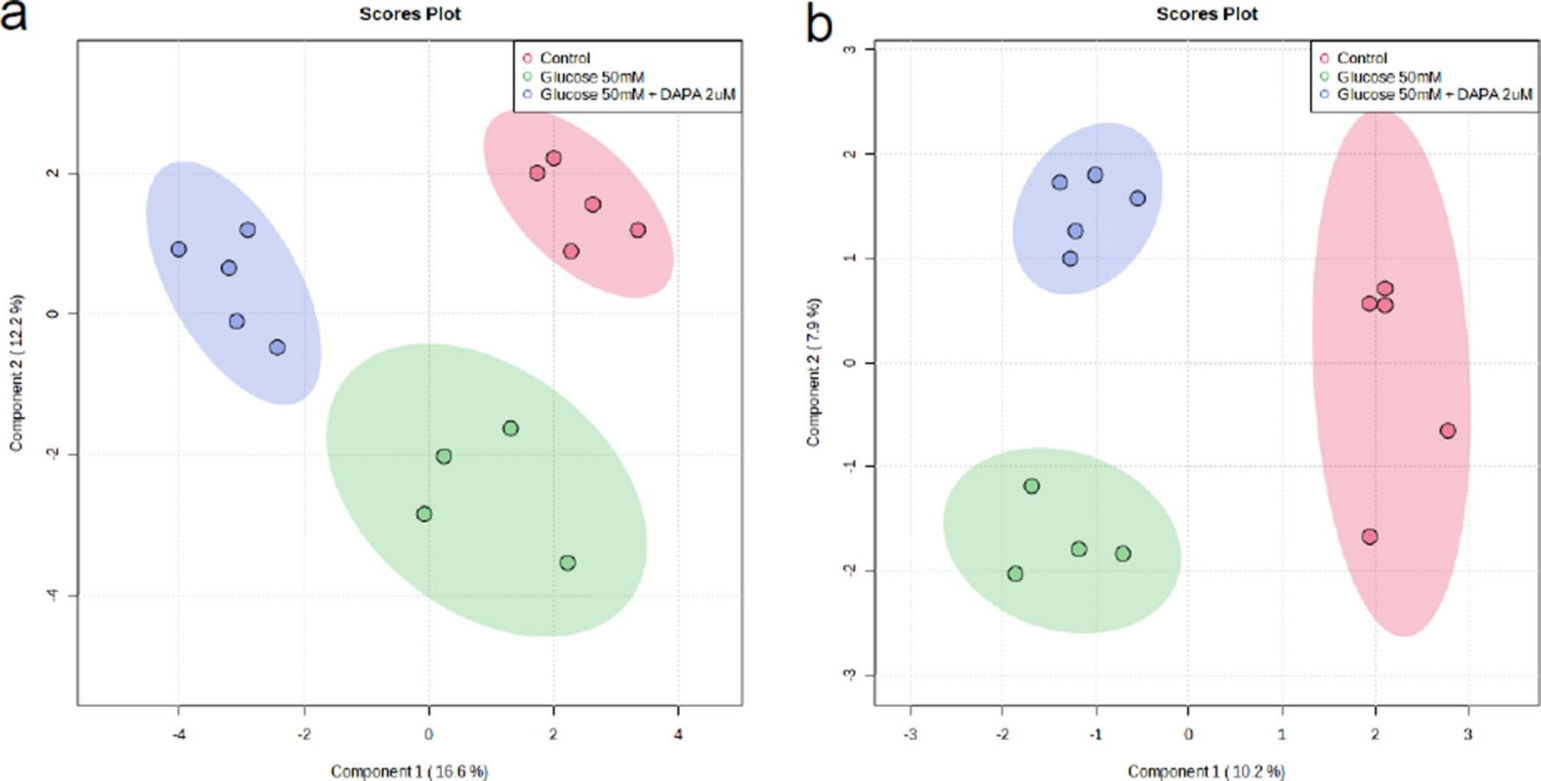
Sparse partial-least-squares discriminant analysis (sPLS-DA) of metabolites detected in tubular epithelial cells (**a**), podocytes (**b**), respectively, treated with 2 µM dapagliflozin following 50 mM glucose compared to those without treatment (control) and compared to those treated with 50 mM glucose. sPLS-DA in 2D scoring plot with 95% confidence interval.

The metabolomic profiles of tubular epithelial cells and podocytes under high glucose conditions (25 and 50 mM glucose) were compared using sPLS-DA and heatmap (Figs. 1 and S1). The metabolomic pattern of tubular epithelial cells was discriminated between 50 mM glucose-treated group and the control. The metabolomic pattern of podocytes was distinctly separated between the 25 mM or 50 mM glucose-treated group and the control in the sPLS-DA. The metabolomic patterns in tubular epithelial cells, podocytes, respectively, treated with 2 µM dapagliflozin following high glucose stimulus (50 mM glucose) compared with those without treatment and compared to those treated with 50 mM glucose was differentiated using sPLS-DA and a heatmap (Figs. 2 and S2).

### Changes in the metabolomic profile of tubular epithelial cells treated with SGLT2 inhibitor following high glucose stimulus

**Figure 3:**
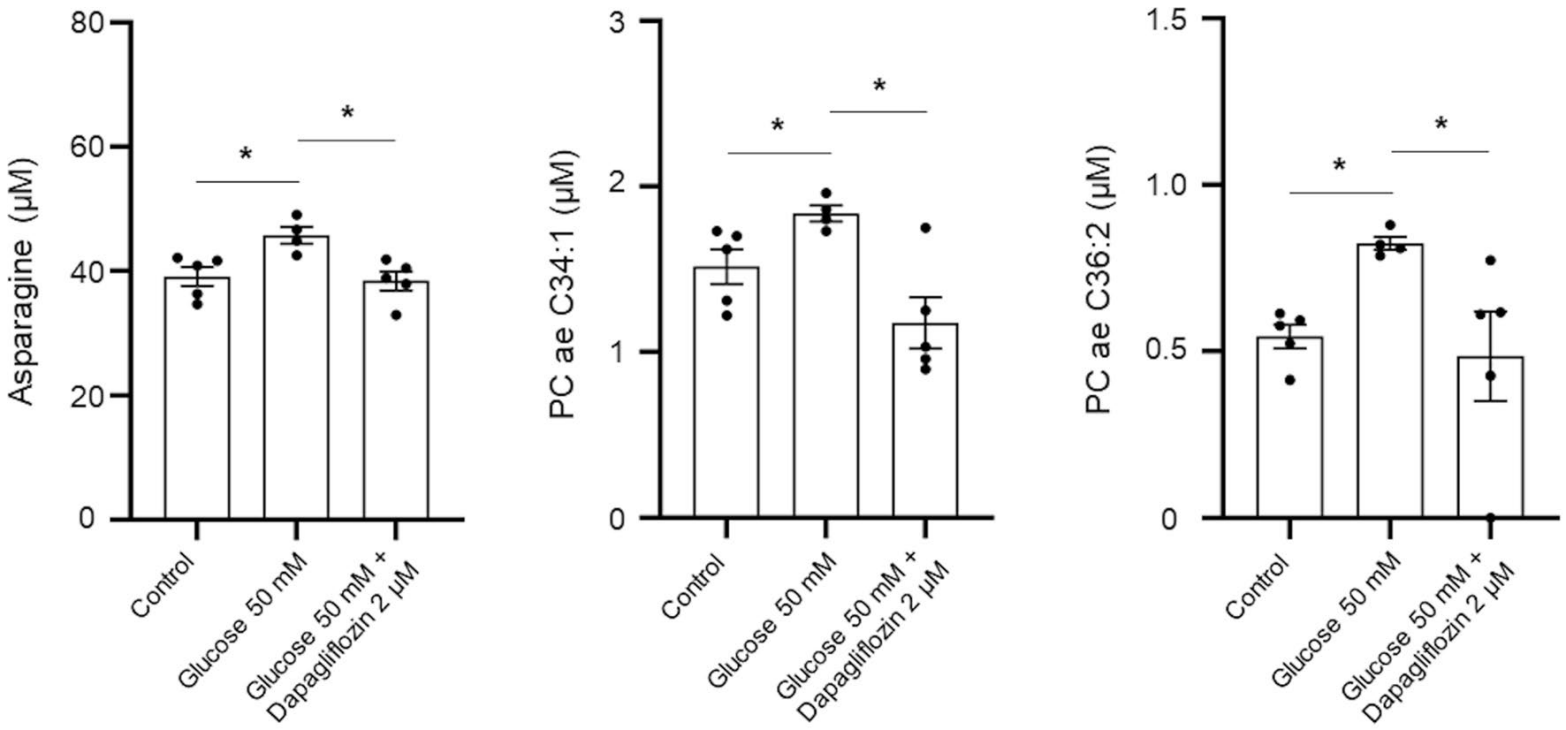
Box plots of differentially expressed metabolite levels in tubular epithelial cells treated with 2 µM dapagliflozin following 50 mM glucose compared to the levels of metabolite identified in those without treatment or treated with 50 mM glucose. *p < 0.05 indicates the significant difference between the groups by unpaired nonparametric Mann–Whitney test.

The level of C16, PC ae C36:2, PC ae C34:1, and asparagine was increased in tubular epithelial cells treated with 50 mM glucose compared to those without treatment (Table S1). Conversely, the metabolite level of spermidine and putrescine was decreased in tubular epithelial cells treated with glucose 50 mM compared to those without treatment. The tubular epithelial cells treated with dapagliflozin showed a significantly decreased level of 27 metabolites (ornithine, glycine, PC ae C30:0, isoleucine, and PC ae C34:2, PC aa C32:0, PC ae C36:2, PC aa C38:4, PC aa C34:2, PC ae C34:1, PC aa C36:2, spermidine, lysine, histidine, arginine, PC aa C36:3, alanine, PC aa C34:1, spermidine, serine, valine, phenylalanine, proline, putrescine, threonine, tyrosine, and asparagine) compared with those treated with 50 mM glucose (Table S2). Of these metabolites, the levels of asparagine, PC ae C34:1, and PC ae C36:2 were elevated in tubular epithelial cells stimulated with 50 mM glucose but significantly decreased after dapagliflozin treatment (Fig. 3).

### Changes in the metabolomic profile of podocytes treated with SGLT2 inhibitor following high glucose stimulus

**Figure 4:**
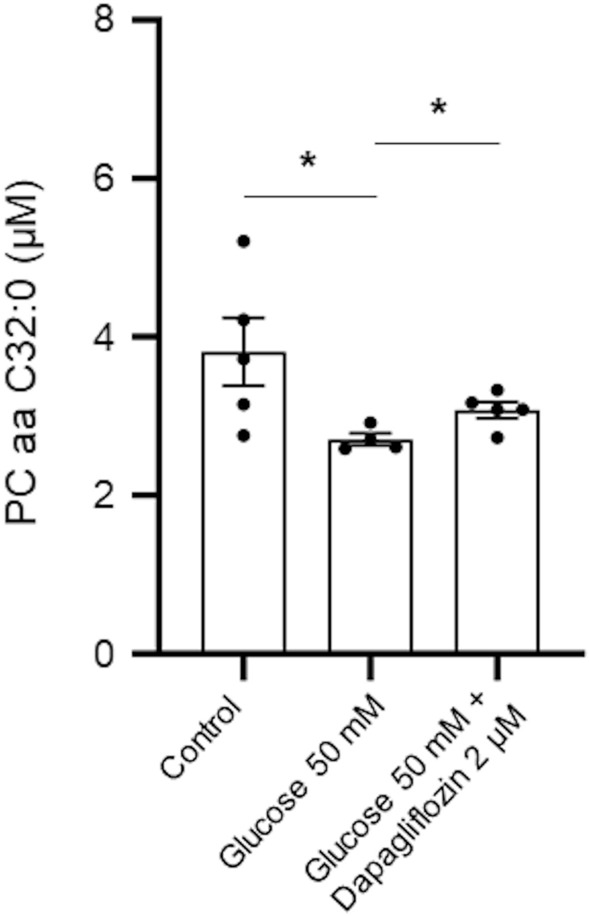
Box plots of differentially expressed metabolite levels in podocytes treated with 2 µM dapagliflozin following 50 mM glucose compared to the level of metabolite identified in those without treatment or treated with 50 mM glucose. *p < 0.05 indicates the significant difference between the groups by unpaired nonparametric Mann–Whitney test.

The level of five metabolites (H1, C14:1-OH, putrescine, proline, and spermidine) was increased, while the level of PC aa C32:0 and PC aa C34:3 was decreased in podocytes treated with 50 mM of glucose compared to those without treatment (Table S3). Of these metabolites, the level of PC aa C32:0 was significantly decreased after 50 mM glucose treatment compared to those without treatment, while its level was significantly increased after dapagliflozin treatment (Table S4, Fig. 4).

### Pathway analysis in the metabolomic profile of tubular epithelial cells

**Figure 5:**
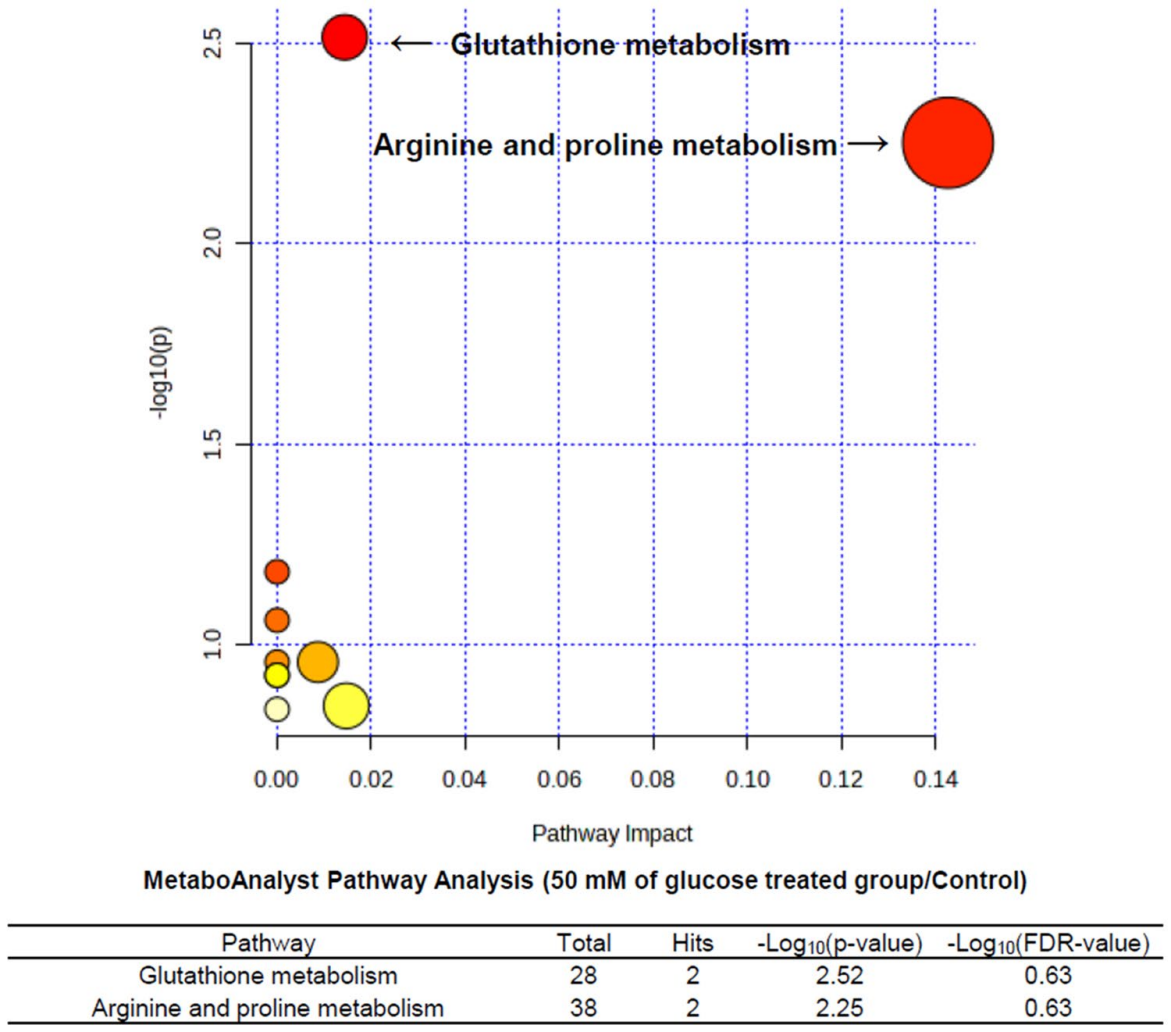
The metabolic pathways analysis for metabolites expressed in tubular epithelial cells treated with 50 mM of glucose compared to those without treatment (control) was performed using MetaboAnalyst. All the matched pathways in KEGG database are displayed as circles. The color and size of each circle represents *p* value and pathway impact value, respectively.

**Figure 6:**
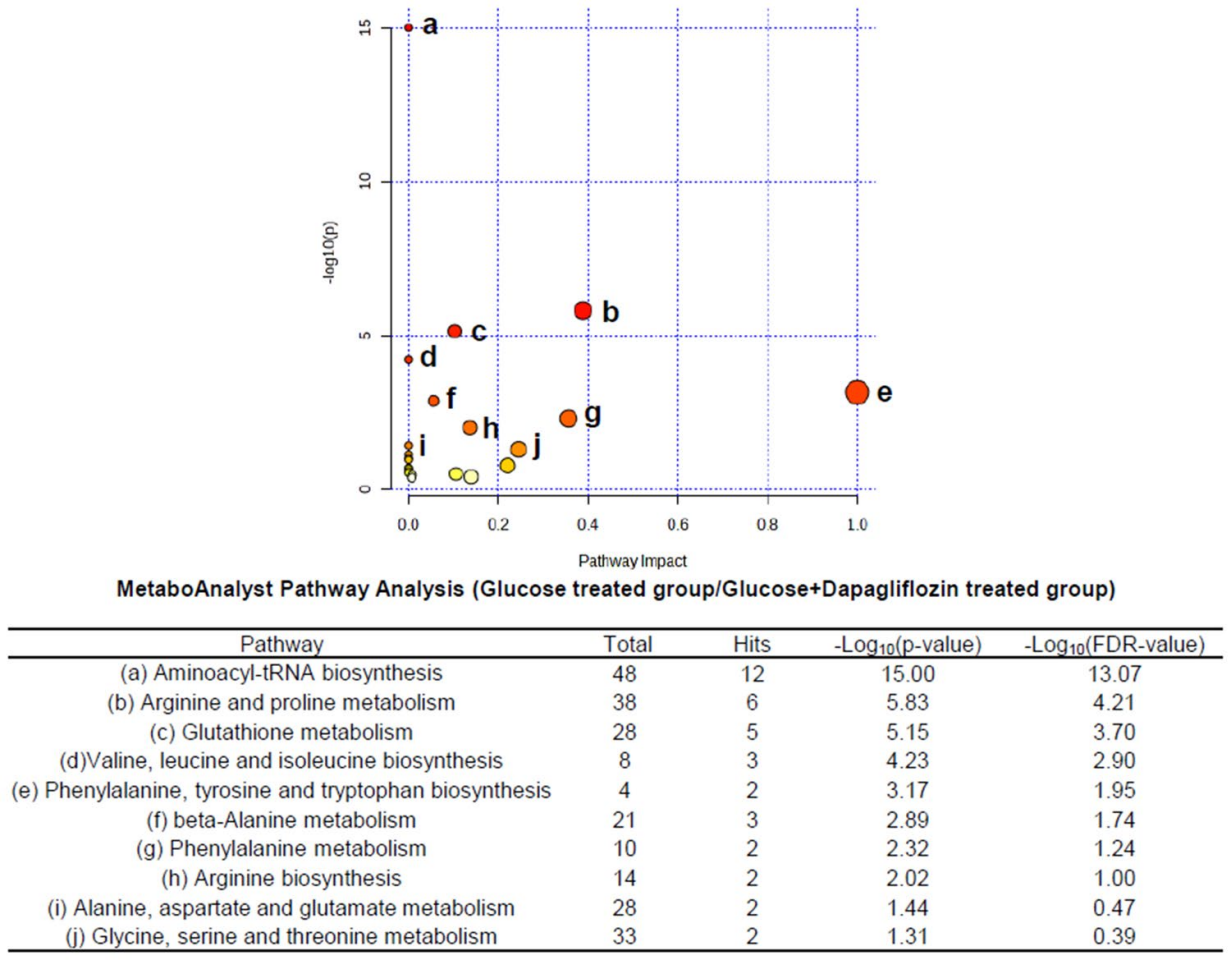
The metabolic pathways analysis for metabolites expressed in tubular epithelial cells treated with 2 µM dapagliflozin following 50 mM glucose compared to the level of metabolite identified in those treated with 50 mM glucose was performed using MetaboAnalyst. All the matched pathways in KEGG database are displayed as circles. The color and size of each circle represents *p* value and pathway impact value, respectively.

MetaboAnalysis pathway analysis was done using metabolites with *p* value < 0.05 in KEGG database. The metabolism of glutathione, asparagine and proline was significantly changed in the 50 mM of the glucose-treated tubular epithelial cells compared to those without treatment (Fig. 5). Also, the differences of aminoacyl-tRNA biosynthesis, arginine and proline metabolism, glutathione metabolism, valine, leucine and isoleucine biosynthesis, phenylalanine, tyrosine, and tryptophan biosynthesis, beta-alanine metabolism, phenylalanine metabolism, arginine biosynthesis, alanine, aspartate and glutamate metabolism, glycine, serine and threonine metabolism were noted in tubular epithelial cells after treatment of dapagliflozin compared to those treated with 50 mM of glucose (Fig. 6).

## Discussion

The metabolomic analysis using primary cultured kidney cells under high glucose conditions has not been investigated although the study using only human tubular epithelial cell lines has been performed^10,18^. Therefore, this study aimed to investigate the metabolomic profile of primary cultured human kidney cells under high glucose conditions and the pattern changed in response to dapagliflozin, which is an SGLT2 inhibitor. Furthermore, the diabetic nephropathy milieu comprises a complicated signaling network that connects various types of kidney cells via secretory mediators^9^. Therefore, the combined metabolomic analysis at the cellular levels of the different types of kidney cells under diabetic conditions following SGLT2 inhibitor treatment can be an insightful tool to provide clues regarding cellular signaling caused by the pleiotropic effects of the SGLT2 inhibitor.

A significant change in metabolite profiling was evident in tubular epithelial cells treated with an SGLT2 inhibitor, presumably due to the immediate alteration in these cells as the target of SGLT2 inhibitors. The levels of asparagine, PC ae C34:1, and PC ae C36:2 were increased by high glucose stimulation, and the levels of these metabolites were reversed by treatment with dapagliflozin in tubular epithelial cells. Asparagine is the gluconeogenic amino acid used as the substrate for the anaplerosis of tricarboxylic acid cycle and is essential for cellular growth in glutamine-depleted circumstances^19^. Asparagine has been correlated with homeostatic model assessment for insulin resistance in diabetes patients^20^, and a previous metabolomic study found that elevation of its level in the serum of the population has an inverse relationship with the progression of diabetes risk^21,22^. It has been previously identified that there was a positive correlation between the change in body weight and the concentration of amino acids, including asparagine, in patients with diabetes mellitus treated with dapagliflozin compared with a control group using serum metabolomic analysis^23^. It has been shown that the relief of mitochondrial stress through inhibiting the electron transport chain has prevented tumor growth by lowering intracellular asparagine^24^. The present study used dapagliflozin at a concentration of 2 µM which has proven to inhibit the endoplasmic reticulum stress and the expression of activating transcription factor 4 in the kidney tubular cell lines^25^. Although further studies need to be investigated, asparagine synthetase which of the target of activating transcription factor 4^24^ that has been proven to be inhibited by dapagliflozin^25^ can explanation the attenuation of asparagine level by dapagliflozin in the tubular cells.

Regarding phosphatidylcholine, the levels of two metabolites, PC ae C34:1 and PC ae C36:2, which are acyl-alkyl phosphatidylcholines, increased in tubular epithelial cells following high-glucose stimulation and decreased with dapagliflozin treatment. Previous studies have shown that increased phosphatidylcholine level in the serum of patients with hypertension and PC ae C34:1 was positively correlated with hypertension^26,27^. PC ae C36:2 was known to be correlated with diabetes mellitus risk using serum metabolomic in a Korean diabetes cohort^28^. Altered phosphatidylcholine metabolism was linked with a meal, insulin resistance, and diabetes, and acyl-alkyl phosphatidylcholine is known to act as an antioxidant during lipid oxidation^29,30^.

Among the metabolites that were downregulated in podocytes treated with glucose, only PC aa C32:0, a diacyl-phosphatidylcholine, was reversed with dapagliflozin treatment. Diacyl-phosphatidylcholine is positively correlated with an increased risk of diabetes^26^. Contrary to the previous study, in the present study using podocytes, the cellular level of PC aa C32:0 was attenuated under diabetic conditions.

Proximal tubular epithelial cells rely on their energy demand by using fatty acid oxidation rather than glycolysis, and glucose in the proximal tubular epithelial cell is shunted into the pentose phosphate pathway that can maintain antioxidant glutathione in its reduced state in response to injury^31^. Enhanced oxidative stress, along with increased metabolites of the tricyclic acid cycle upon high-glucose stimulation, resulted in altered glutathione metabolism in diabetic kidney tissue^32^. The altered glutathione metabolism in the tubular epithelial cell upon high-glucose treatment might originate from antioxidant responses to increased oxidative stress under diabetic conditions.

The metabolomic study using the mouse renal cortical tissue has shown that differences in metabolism are evident in the ischemia/reperfusion + dapagliflozin group compared with the ischemia/reperfusion group [aminoacyl-tRNA biosynthesis, arginine and proline metabolism, valine, leucine and isoleucine biosynthesis, phenylalanine, tyrosine, and tryptophan biosynthesis, alanine, aspartate and glutamate metabolism, glycine, serine and threonine metabolism]^33^. Also, the significantly altered pathway of aminoacyl-tRNA biosynthesis, arginine and proline metabolism, bet-alanine metabolism has been identified in a diabetic mouse model after SGLT2 intervention^34^.

The renal arginine metabolism mainly occurs in proximal tubular epithelial cells^35^, and the previous study has shown that exposure to albumin can enhance arginine metabolism in these cells^36^. Arginase II is localized in the mitochondria and is mainly expressed in the kidney, which hydrolyzes the arginine to ornithine and then it can be converted into proline^35^. Arginase competes with nitric oxide synthase, which uses arginine as its substrate, and the decreased arginase activity eventually means an increase in nitric oxide combined with protective effect in the ischemic injury^37^. Furthermore, the increased activity of arginase has evident in the ischemia/reperfusion injury in the renal tubules and also in proximal tubular cell lines in hypoxia/reoxygenation injury model^38^. The inhibition of arginase has shown a protective effect on tubular epithelial cells, which has increased oxygen stress^38^. Although further study needs to investigate, we suggest that cellular metabolism might have directional for protective nitric oxide based on the results of the dapagliflozin effect on the decreased level of metabolites, including arginine, ornithine, and proline in the tubular epithelial cells. The pathognomic feature of diabetic nephropathy results from various inflammatory processes caused by high-glucose-induced oxidative stress and the accumulation of extracellular matrix in kidney tissues. The combined effect of inflammatory responses to high glucose in various cells will appear in the in vitro metabolomics study. The increased production of oxidative stress induced by high glucose is capable of causing oxidative damage to proteins, carbohydrates, or lipids in the cells^39^.

Since diabetic kidney disease is an inflammatory response mediated by secretory mediators through cell-to-cell crosstalk in various kidney cells, our study was limited in that we could not investigate the metabolomic profiling of a coculture system. Therefore, a comprehensive analysis by performing an integrated metabolomic study is required to detect metabolites that can be changed by secretory mediators from the various kidney cells under high glucose conditions with SGLT2 inhibitor treatment. Another limitation of this study is that we used targeted metabolomics analysis, which was unable to detect TCA cycle intermediates that might be altered in kidney cells under high glucose conditions^40^, and we could not perform lipidomics.

Despite the limitation of metabolomic study in vitro, the altered metabolism of glutathione in the tubular epithelial cells might have derived from increased oxidative stress in the tubular epithelial cells treated with high-glucose. Altered metabolites of asparagine, phosphatidylcholine, and aminoacyl tRNA biosynthesis, arginine and proline metabolism have to be further investigated to identify protective effect of SGLT2 inhibitor.

## Supplementary Information

Supplementary Information.

## Data Availability

The data generated during and/or analyzed during the current study are included in this article and its supplementary information files.
