# Supplementary material for: Metabolomic profiling in kidney cells treated with a sodium glucose-cotransporter 2 inhibitor
Source: Sci Rep. 2023 Feb 4;13:2026. doi: 10.1038/s41598-023-28850-3 (PMC9899225; doi:10.1038/s41598-023-28850-3)

Table S1. Metabolites differentially expressed in tubular epithelial cells treated with 50 mM glucose compared to those without treatment.

	Glucose 50 mM vs. Control	
Metabolites	Fold difference	*p*-value
C16	2.46	0.024
PC ae C36:2	0.60	0.016
PC ae C34:1	0.28	0.024
Asparagine	0.23	0.016
Spermidine	-0.29	0.016
Putrescine	-0.59	0.016

ae, acyl-alkyl. The data were analyzed using the Mann-Whitney test.

Table S2. Metabolites differentially expressed in tubular epithelial cells treated with 2 µM dapagliflozin following 50 mM glucose compared to those treated with 50 mM glucose.

	Glucose 50 mM vs. Glucose 50 mM + Dapagliflozin 2 µM	
Metabolites	Fold difference	*p* -value
Ornithine	2.81	0.024
Glycine	1.45	0.016
PC ae C30:0	1.43	0.016
Isoleucine	1.34	0.016
PC ae C34:2	1.29	0.016
PC aa C32:0	0.79	0.016
PC ae C36:2	0.76	0.016
PC aa C38:4	0.68	0.016
PC aa C34:2	0.65	0.016
PC ae C34:1	0.64	0.032
PC aa C36:2	0.62	0.016
Spermidine	0.58	0.016
Lysine	0.56	0.016
Histidine	0.56	0.016
Arginine	0.55	0.016
PC aa C36:3	0.54	0.016
Alanine	0.52	0.016
PC aa C34:1	0.52	0.016
Spermine	0.51	0.016
Serine	0.49	0.016
Valine	0.49	0.016
Phenylalanine	0.49	0.016
Proline	0.48	0.016
Putrescine	0.45	0.016
Threonine	0.37	0.016
Tyrosine	0.34	0.032
Asparagine	0.25	0.016

ae, acyl-alkyl; aa, acyl-acyl. The data were analyzed using the Mann-Whitney test.

Table S3. Metabolites differentially expressed in podocytes treated with 50 mM glucose compared to those without treatment.

	Glucose 50 mM vs. Control	
Metabolites	Fold difference	*p*-value
H1	2.77	0.016
C14:1-OH	2.39	0.032
Putrescine	1.07	0.016
Proline	0.36	0.016
Spermidine	0.33	0.016
PC aa C32:0	-0.50	0.032
PC aa C34:3	-1.01	0.032

OH, hydroxyl; aa, acyl-acyl. The data were analyzed using the Mann-Whitney test.

Table S4. Metabolites differentially expressed in podocytes treated with 2 μM dapagliflozin following 50 mM glucose compared to those treated with 50 mM glucose.

	Glucose 50 mM vs. Glucose 50 mM + Dapagliflozin 2 μM	
Metabolites	Fold difference	*p*-value
PC aa C32:0	-0.19	0.024

aa, acyl-acyl. The data were analyzed using the Mann-Whitney test.

Figure S1. Heatmap of metabolites detected in tubular epithelial cells (a), podocytes (b), respectively, treated with glucose (25, 50 mM) compared with control.

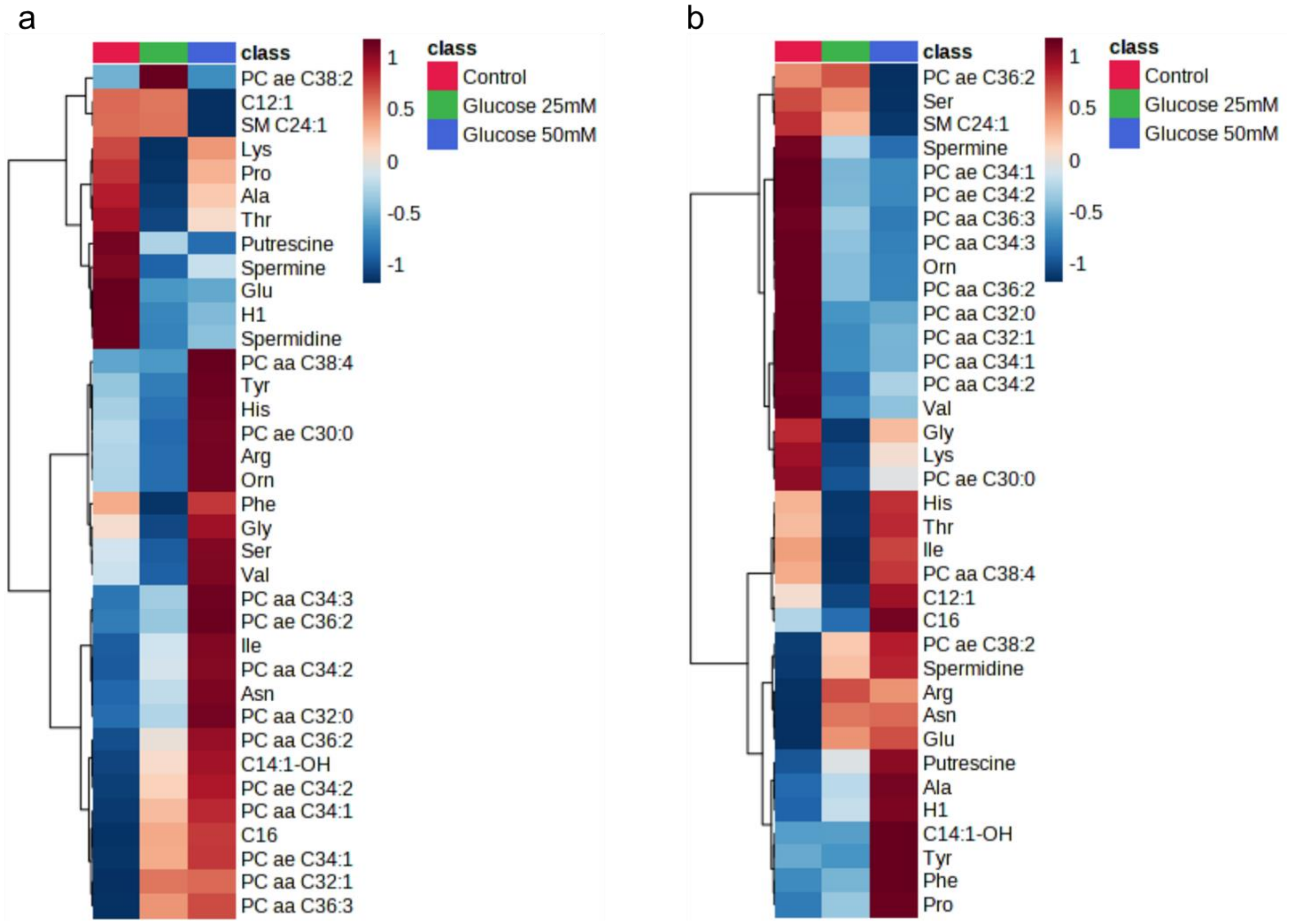

Figure S2. Heatmap of metabolites detected in tubular epithelial cells (a), podocytes (b), respectively, treated with 2 μM dapagliflozin following 50 mM glucose compared with those without treatment and compared to those treated with 50 mM glucose.

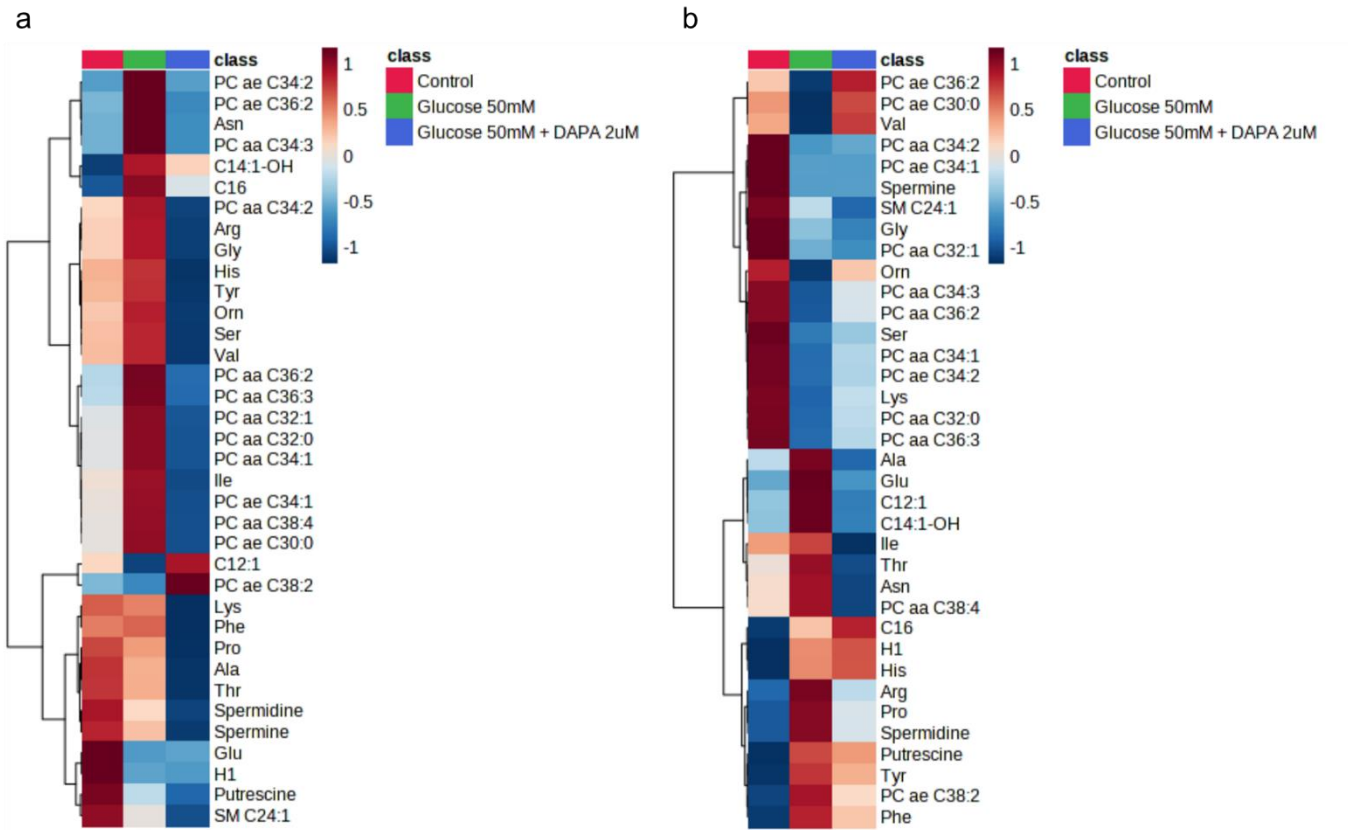

Supplement: Supplementary file 1 — Supplementary Information. [file 41598_2023_28850_MOESM1_ESM.pdf]
